# A Novel Case Report of Severe Aplastic Anemia with COVID Infection

**DOI:** 10.4314/ejhs.v33i1.22

**Published:** 2023-01

**Authors:** Fatemeh Nejatifar, Ezat Hesni, Ali Akbar Samadani

**Affiliations:** 1 Department of Hematology and Oncology, Guilan University of Medical Sciences, Rasht, Iran; 2 Department of infectious diseases , Guilan University of Medical Sciences, Rasht, Iran; 3 Guilan Road Trauma Research Center, Guilan University of Medical Sciences, Rasht, Iran

**Keywords:** Severe aplastic anemia, hematopoietic system, COVID-19, supportive treatment

## Abstract

Aplastic anemia is a rare disease of the hematopoietic system. Although some viral agents have been implicated, the association between COVID-19 and aplastic anemia is unclear. In this way, several cases of aplastic anemia have been reported following infection with COVID-19. Importantly, we reported a 16-year-old girl with severe aplastic anemia with no history of disease following an Omicron infection who did not respond well to treatment despite supportive treatment and immunosuppression.

## Introduction

Aplastic anemia (AA) is a seldom hematological position that is indicated by the main pancytopenia because of bone marrow failure. In this way, it is confirmed that this type of special bone marrow failure syndrome is the result of the destruction of hematopoietic stem cells (HPSC) that is caused due to the dysregulated auto-immune signal. For the etiology of this disease, we can say that ionizing radiation, chemotherapy, and also viral infections are associated with this disease (1).

Although COVID has more pulmonary manifestations, involvement of various organs including the hematopoietic system has been reported. Hematologic disorders include Lymphopenia, thrombocytopenia, anemia, and other common complications. It is noteworthy that the relationship between aplastic anemia and COVID is still unknown. (2, 3). In this case report, we explain a rare case of severe aplastic anemia with the COVID-19 virus.

## Case Presentation

The patient was a 16-year-old woman with severe weakness, lethargy, and blurred vision during the sixth peak of COVID-19 (Omicron) in Iran. In this way, she had a history of fever and chills with myalgia and loss of appetite, sore throat, and dry cough in the last week. She had also, a mild fever with chills and dry cough without hemoptysis and epistaxis with no evidence of bleeding mentioned. Remarkably, it did not indicate shortness of breath, chest discomfort, or chest pain. There was also disclosed contact history of a suspect with COVID-19 family members. The second dose of the Sinopharm vaccine was injected one week before the disease. She has been receiving Symptomatic treatment at home for a week due to fever and cough, and she has been referred to the center due to the aggravation of her symptoms and severe weakness, bullerd vision and menorrhagia, and also lethargy. During the initial emergency examination, severe pancytopenia was diagnosed and the patient was admitted to the center. Conspicuously, no history of previous illness, medication, recent travel, contact with animals, or tick bites was reported. Clinical examination detected slight erythema on throat examination without exudate and mucosal lesions or Petechiae, and evidence of bleeding in the mouth and gums. The pale conjunctiva was seen without jaundice and evidence of bleeding. Normal abdominal and pelvic examination was diagnosed without organomegaly and lymphadenopathy. Retinal hemorrhage was seen in both eyes and was preferred on the right eye examination. Vital signs were included like Bp(blood pressure)80 /40 mmHg, PR(pulse rate)113 beats per minute, RR(respiratory rate)18 breaths per minute, BT(body temperature)36.5c^0^, o2sat 100 % and preliminary tests were comprised Wbc=800 10 ^3^ /microliter, Hbg=3.9 g/dl, Plt =6000 /10 ^3^ /microliter, Esr= 135 mm/hour, Procalcitonin 30 ng/ml. Additionally, other tests were done due to severe Neutropenia and Systolic blood pressure of 80 with Tachycardia. Meanwhile, the blood and urine cultures were sent to the laboratory for more consideration (Table 1).

**Table 1:** Important variables that were measured

Variable	DAY1	DAY9	DAY14	Normal Range
blood sugar(mg/dl)	151			
Bun(mg/dl)	10			7–21
Creatinine(mg/dl)	0.96			0.6–1.3
Bilirubin(Total) (mg/dl)	0.5			0.3–1.2
Bilirubin(Direct) (mg/dl)	0.2			0–0.2
SGOT(U/L)	13			<41
SGPT(U/L)	30			<41
Alkaline phosphatase(U/L)	342			70–290
CPK(U/L)	20			>140
LDH(U/L)	210			240–480
Amylase(U/L)	12			<90
Phosphorus(mg/dl)	4.4			2.7–4.5
Na(mEq/l)	139			135–145
K(mEq/l)	3.7			3.5–5.3
MAGNESIUM(mg/dl)	2.8			1.53–2.55
Lipase(U/L)	17			<=60
CRP(mg/L)	207			<10
White-cell count(x10^3/UL)	0.8	2.7	2.6	4–11
Red-cell count (x10^3/UL)	1.33	2.62	2.87	4.5–5.1
Hemogelobin(g/dL)	3.9	7.9	8.5	12.3–15.3
Heamatocrit%	13.3	25.3	27.3	34.5–44.6
M.C.V(FL)	100	96.6	95.1	80–100
M.C.H(pg)	29.3	30.2	29.6	
M.C.H.C(gr/dl)	29.3	31.2	31.1	31–37
Platelet(x10^3/UL)	6	53	27	150–450
Neutrophil%		9	7	
Lymphocyte%		89	90	
Monocyte%		2	2	
Anisocytosis	+			
Hypochromia	+			
ESR 1hr(mm/hr)	135			Up to 30
VBG	110			90–110
Chloride(mEq/l)	7.4			
PH	39.5			
Pco2(mmHg)	21			
Po2(mmHg)	24.7			
Hco3(mEq/l)	35.5			95–100
O2 Sat%	_0.1			_2 to +2
Be(mmol/L)	0			
BB(mmol/L)	13.3			11–13.5
PT(Sec)	12			
Protrombin.control(Sec)	1.2			1–1.3
INR	28			28–40
PTT(Sec)				
Urinanalysis				
Urinanalysis	Yellow			
Color	Semi Clear			
Appearance	1010			
Specific gravit	7			
PH	Neg			
Urine protein	Trace			
Glucose	Neg			
Bilirubin	Neg			
Urobilinogen	Neg			
Keton	1+			
Blood	2–3			
White-cell count(perµgl)(x10^3/UL)	8–10			
Red-cell count(perµgl)) (x10^3/UL)	6–8			
Epithelial cell	Neg			
Nitrite	No growth			
Urine culture & Sensitivity	E.coli			
Blood Culture X2	0.3			Up to1.5
Reticulocyte count	158			200–400
Fibrinogen	674			

VitaminB12(pg/ml)				

**Figure 1:**
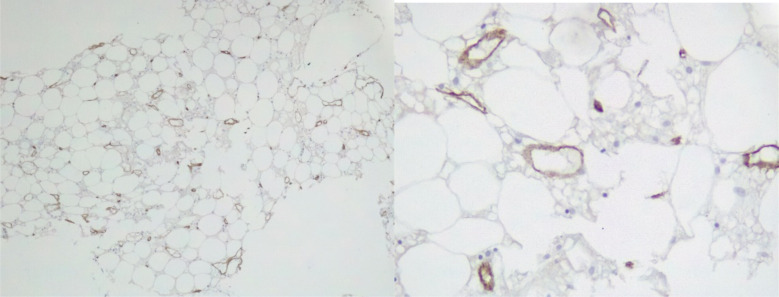
Immunohistochemical stains reveal decreased number of cd34 cells

**Figure 2:**
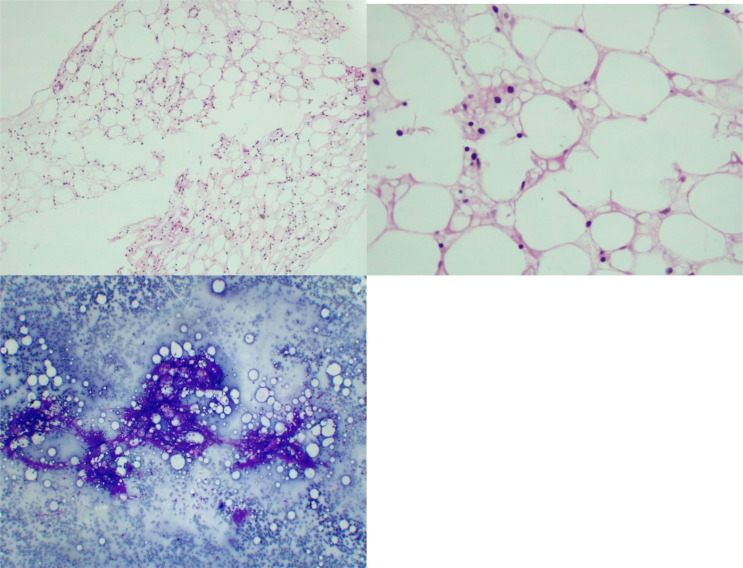
Biopsy and aspiration of bone marrow show severe hypocellular marrow for age.

In this account, broad-spectrum antibiotics including Meropenem and Ciprofloxacin were started together with Vancomycin. Relatively, CXR(chest x-ray) and abdominal and pelvic ultrasound were performed. On the X-ray of the chest, a slight increase in bilateral broncho vascular marking without consolidation was observed and the COVID-19 RT-PCR was positive. The patient was treated with Remdesivir 200 mg initial dose and continued 100 mg daily for 5 days with Dexamethasone 6 mg daily. Supportive treatment including packed cell, blood, and platelet transfusion was performed due to retinal bleeding. bone marrow aspiration and biopsy were done. In the bone marrow study, cellularity decreased by 5-10% without increasing blast and CD 34 was less than 1 percent (Figures 1 and 2). In flow cytometry of bone marrow, severely hypocellular marrow and normal B- and T cells were reported. Secondary causes of aplastic anemia including hepatitis B, C, HIV, CMV, Parvovirus b19, and EBV were reported negative. After completing COVID-19 treatment, treatment with cyclosporin 3 mg per kg, Romiplostim, and erythropoietin was started. The patient was discharged in good general condition and was followed up on an outpatient basis. Correspondingly, the patient was a candidate for bone marrow allogeneic transplantation and the donor did not have a proper sibling and the search for an unrelated donor began. In most COVID cases, pancytopenia is transient and mild and does not require bone marrow examination.

## Discussion

COVID-19, which is caused by the SARS-COVID virus, has been an epidemic since 2019 and causes a mild to fatal disease that has multisystem symptoms. Hematologic disorders are common in this disease. One of the rare cases of aplastic anemia is viral causes. Various mechanisms have been proposed for the occurrence of bone marrow failure in viral infections, including destabilizing and increasing the production of inflammatory cytokines and destruction of hematopoietic cells by the virus. In this account, some viruses have been linked to this disease, but the association between COVID and aplastic anemia is not yet known (4). In most cases, pancytopenia is transient and mild and does not require bone marrow examination. Vikram et al., reported a 29-year-old woman with a history of seizures, developing aplastic anemia after COVID, which responded after several courses of immunosuppressive therapy. Our patient did not respond well to treatment and needed a blood transfusion and platelets. Ranjima et al. reported a 4-year-old girl who developed aplastic anemia after COVID, Which did not satisfactorily respond to treatment and became a candidate for bone marrow transplantation (5). A 25-year-old woman with a history of primary glomerulonephritis, who developed aplastic anemia after COVID, did not respond well after two months of immunosuppressive therapy and became a candidate for a bone marrow transplant. Our patient, without a history of any specific disease, developed severe aplastic anemia after COVID-19, which did not respond well to supportive and immunosuppressive therapy. A 53-year-old man with a history of mantle-cell lymphoma who underwent autologous bone marrow transplantation and is currently receiving monoclonal antibody treatment was admitted with pancytopenia and severe respiratory distress. After 45 days, COVID PCR was positive in peripheral blood and bone marrow. Antiviral therapy was used to treat pancytopenia. The cause of pancytopenia was explained to be bone marrow infection with the virus. COVID-19 can cause hematologic symptoms such as thrombocytopenia and lymphopenia, anemia, but giving pancytopenia and reducing cellularity by up to 5% is rare and few cases have been reported. The pathogenesis of COVID in the development of aplastic anemia and the course and mediation of these patients is not completely clear.

## References

[1] Miano M, Dufour C (2015). The diagnosis and treatment of aplastic anemia: a review. International journal of hematology.

[2] Ziegler CG, Allon SJ, Nyquist SK, Mbano IM, Miao VN, Tzouanas CN (2020). SARS-CoV-2 receptor ACE2 is an interferon-stimulated gene in human airway epithelial cells and is detected in specific cell subsets across tissues. Cell.

[3] Nejatifar F, Rostami S, Chahardouli B, Kasaeian A, Vaezi M, Kamranzadeh H (2022). Incidence and Prognostic Impact of WT-1 Gene Exon7 and 9 Mutations in Acute Promyelocytic Leukemia. International Journal of Hematology-Oncology and Stem Cell Research.

[4] Nejat N, Jadidi A, Hezave AK, Pour SMA (2021). Prevention and treatment of COVID-19 using traditional and folk medicine: A content analysis study. Ethiopian Journal of Health Sciences.

[5] Ranjima M, Gobbur R (2021). Severe Aplastic Anemia Secondary to SARS CoV-2 Infection-A Case Report. Journal of Pediatrics, Perinatology and Child Health.

